# A qualitative analysis of nicotine replacement therapy uptake, consistent use, and persistence among primary care patients who smoke

**DOI:** 10.1016/j.dadr.2021.100018

**Published:** 2021-12-11

**Authors:** GM Styklunas, NN Shahid, ER Park, JE Haberer, NA Rigotti, SE Howard, GR Kruse

**Affiliations:** a Division of General Internal Medicine, Massachusetts General Hospital, Boston, MA, United States; b Tobacco Research and Treatment Center, Massachusetts General Hospital, Boston, MA, United States; c Tufts University School of Medicine, Boston, MA, United States; d Department of Psychology, University of Miami, Miami, FL, United States; e Center for Global Health, Massachusetts General Hospital, Boston, MA, United States; f Harvard Medical School, Boston, MA, United States; g Health Policy Research Center, Department of Medicine, Massachusetts General Hospital, Boston, MA, United States

## Abstract

• Adherence can be broken into three processes: uptake, consistent use, and persistence.
• Barriers and facilitators to NRT use vary over the three adherence processes.
• Information gaps and negative stories about NRT are common barriers to adherence.
• NRT adherence may be improved by addressing patient knowledge and concerns.

## 1 Introduction

Nicotine replacement therapy (NRT) is the most widely used smoking cessation medication with a robust evidence base demonstrating its efficacy and safety (Anthenelli et al., 2016; Wadgave and Nagesh, 2016). In the US 14–17% of individuals who are trying to quit use some form of NRT (Babb et al., 2017). Compared to placebo, NRT doubles the likelihood of a successful quit attempt (Anthenelli et al., 2016; Raupach et al., 2014; Schlam et al., 2018). However, the effectiveness of NRT in real-world settings has not reached the effectiveness levels found in randomized trials. This is thought to be due, in part, to sub-optimal adherence (Kotz et al., 2014a, 2014b). Up to 20–50% of individuals who smoke and who are given NRT prescriptions never fill them (Kim et al., 2019; Solberg et al., 2010), and less than 40% adhere to the recommended number of doses each day (Schlam et al., 2018; Yingst et al., 2015). Previous studies have found that barriers to NRT adherence include negative beliefs about NRT safety (Edwards et al., 2021; Kim et al., 2019; McDaid et al., 2021), side effects, desire to quit unassisted (Morphett et al., 2015), NRT affordability, low self-efficacy, resuming smoking, NRT not addressing the behavioral component of smoking (Edwards et al., 2021), and forgetting (Yingst et al., 2015). Friends and family are often the source of information about smoking cessation (Sharma‐Kumar, Meurk, Ford, Beere, and Gartner, 2018).

While several studies have examined the correlates of cessation medication adherence (Pacek et al., 2018), there has been limited success with interventions designed to improve smoking cessation adherence (Hollands et al., 2019). This limited success may be in part due to an incomplete conceptualization of medication adherence behaviors for NRT. Medication adherence has been conceptualized as a multidimensional behavior including: (1) uptake: the primary initiation of medication use; (2) consistent use: the adherence to an accurate and consistent dosing regimen; and (3) persistence: the duration of adherence over a longer period of time (Stirratt et al., 2018). Applying this adherence pathway to NRT use behaviors may allow providers to account for the differences between patients who never start using NRT, do not take the prescribed dosage, and those who discontinue their medication early. Recent literature has also made the case for considering medication adherence as a dynamic rather than static variable (Stirratt et al., 2018), comprised of multiple behaviors that may vary within an individual over time (Stirratt et al., 2018; Vrijens et al., 2012). Our understanding of adherence may be further enhanced by differentiating nonmodifiable factors, such as allergies, from modifiable factors, such as perceptions and self-efficacy (Pacek et al., 2018) within the different dimensions of adherence. Improving the conceptualization of NRT adherence and differentiating each dynamic behavior in the adherence process may allow for more effective identification of targets for NRT adherence promoting interventions.

### 1.1 Theories conceptualizing medication adherence

Currently, nicotine and tobacco research lack a prominent theory for thinking about adherence to NRT and relevant factors for intervention. Several different health behavior theories and frameworks have been applied to tobacco cessation medication adherence, such as the health belief model, motivational interviewing, social cognitive theory, transtheoretical model, self-regulation/common sense model, and the capability, opportunity, motivation, and behavior (COM-B) model (Champion and Skinner, 2008; Conn et al., 2016; DiClemente and Prochaska, 1998; Herbec et al., 2018; Mersha et al., 2020; Noar and Zimmerman, 2005; Willmott et al., 2021). Framing adherence behaviors in theoretical models enables a structured exploration of behavioral mechanisms of this complex activity (Amico et al., 2018). Among the frameworks previously used to guide cessation medication adherence interventions is the information-motivation-behavioral skills (IMB) model of adherence (Fisher et al., 2006). It describes three primary constructs that influence behavior: information and knowledge of the behavior; a person's motivation to perform the behavior; and the behavioral skills necessary to perform the behavior (Rongkavilit et al., 2010). This model has been used to design interventions targeting tobacco cessation medication adherence including NRT adherence (Rivet Amico, 2011; Shelley et al., 2015; Tseng et al., 2017).

### 1.2 Promoting medication adherence in primary healthcare settings

There remains much to learn about how to leverage healthcare systems to improve smoking cessation medication adherence. Only one study has measured the association between healthcare interactions and likelihood of NRT adherence (Solberg et al., 2010). Many preventable factors could be addressed in healthcare settings, such as education about medications and side effects or activities to promote self-efficacy through motivational interviewing or other techniques. Primary care is a key site for delivering smoking cessation assistance, and there is a clear need to understand what factors influence primary care patients’ adherence to NRT. Our primary objective was to examine barriers and facilitators of NRT uptake, consistent use, and persistence, framed in the Information Motivation Behavioral skills theoretical model, among primary care patients who smoke.

## 2 Methods

### 2.1 Design

This study examines qualitative data collected as part of a research program to develop and pilot test a proactive smoking cessation intervention with text messages and NRT among primary care patients in a Massachusetts health care system (Kruse et al., 2018; G. R. Kruse et al., 2020) Semi-structured telephone interviews with patients who smoke or who recently quit and who participated in a feasibility study or a pilot trial were analyzed. Our reporting of qualitative data collection and analysis follows the COREQ guidelines (Tong et al., 2007).

### 2.2 Sampling and participant recruitment

In both the feasibility study and the pilot trial, patients who were seen in the past two years at their primary care practices were proactively contacted about the study. All participants had to (1) be 18 years and older, (2) have a phone that can receive text messages, (3) read and speak English, (4) be a current daily or recent former smoker, (5) have had a primary care visit in the past two years, and (6) not be pregnant or nursing. Participants in the pilot randomized trial also had to have no contraindications to using NRT. In the feasibility study (G. Kruse et al., 2018a) primary care patients were recruited to receive smoking cessation text messages. Fifteen individuals participated in qualitative interviews which examined experiences with the text messages and prior use of NRT (G. Kruse et al., 2018b). The pilot trial randomized 114 participants to receive proactively offered brief telephone advice with text messages and/or mailed NRT (G. R. Kruse et al., 2020). Participants randomized to receive mailed NRT in the pilot trial were offered nicotine patches and/or mint polacrilex lozenges dosed according to package instructions. A purposeful sample of 21 trial participants were invited to participate in a recorded telephone interview at the end of the 12-week trial based on their quit outcome and study arm. Interviews with the 15 participants in the feasibility study who were asked about their prior NRT experiences were combined with the 21 participants who received NRT in the pilot randomized trial to achieve saturation on adherence themes. By combining interviews from both samples, we were able to obtain a more complete picture of adherence processes among primary care patients for the current analysis.

### 2.3 Procedure and interviews

The interviews were completed for the feasibility study between February and April 2017. For the pilot study, interviews were completed between January 2018 and April 2019. Interviews lasted 15–30 min, were audio-recorded, and transcribed verbatim. Study identification numbers were used to link interviews with demographics. In both studies, participants were asked about their decision to use any form of NRT, how they used it, why they discontinued NRT use, and what barriers they may have faced. Interview guides included probes to explore constructs from the IMB model of medication adherence such as reacting to proposed text message content addressing information, motivation or behavioral skills (Fisher et al., 2006). Interviews were completed by the principal investigator (GK) and clinical research coordinators (NS and SEH).

### 2.4 Data analysis

Interview transcripts were analyzed using a framework analysis approach (Ritchie and Spencer, 2002). NVivo12+ was used to organize the data and analysis (QSR Australia). First, all raw data were examined to identify emergent themes and to explore pre-determined themes including the IMB constructs (information, motivation, and behavior) and the three dimensions of the adherence pathway: uptake, consistent use, and persistence. Emergent and *a priori* themes about barriers and facilitators to adherence informed a preliminary first cycle of coding structure. The coding structure was then applied to subsets of five interviews by two coders (NS and GK). The coding structure was refined through discussion with the research team and applied to the next subset of interviews iteratively until the coding structure was finalized, using the constant comparison technique. All transcripts were double coded with the first cycle coding structure with kappas for each theme ranging from 0.72 to 0.96. Coding was at the sentence level and use of multiple codes for a segment of text was permitted. In the second phase of analysis, analytic memos were used to develop descriptive accounts of adherence themes (Miles et al., 2018) and process coding (Saldaña, 2015) was used to explore behavioral processes of NRT uptake, consistent use and persistence (GS and GK).

### 2.5 Ethics

This work (Protocol# 020P001118) was approved by the Massachusetts General Brigham Internal Review Board. All qualitative interviewees participated in a verbal informed consent process conducted by phone; participants were compensated with a $20 gift card for each qualitative interview.

## 3 Results

### 3.1 Participants

Characteristics for the 36 participants are shown in Table 1. Most patients were middle aged, white, with public health insurance, and almost half were female.

**Table 1:** Baseline Characteristics (*n* = 36).

Characteristic	Value*
Age, mean (range), y	51.17 (28–69)
Female sex	16 (44.44)
White race	30 (83.33)
Insurance	
Medicaid	11 (30.56)
Medicare	8 (22.22)
Medicaid & Medicare	6 (16.67)
Private	11 (30.56)

⁎ Data are given as n (%) unless otherwise indicated.

### 3.2 Overview of qualitative findings

We identified 11 major themes (Table 2) represented in one or more of the three stages of medication adherence pathway: uptake, consistent use, and persistence. The themes represented a facilitator or a barrier to use of NRT. Some themes could be seen as either a barrier or facilitator depending on the situation. Two key themes emerged as factors affecting all three adherence processes: peer narratives about NRT and NRT information needs. Frequencies are provided for the number of times this theme was represented for each behavior. Exemplary quotes and the number of participants demonstrating these themes over all three adherence behaviors are shown in Table 3.

**Table 2:** Key Barriers and Facilitators to Nicotine Replacement Therapy Uptake, Consistent Use, and Persistence.

Uptake	Consistent Use	Persistence
Cost		
Readiness to quit		
Negative view of nicotine		
Smoke-free situations		
	Incorporation into routine	
Peer narratives about NRT	Peer narratives about NRT	Peer narratives about NRT
Information gaps	Information gaps	Information gaps
		Disliked taste
		Side effects
		Alleviation of oral fixation
		Experienced effectiveness

**Table 3:** Key barriers and facilitators: exemplary quotes*.

**Cost (*n*** **=** **5)**	**Uptake**
	The lozenges [worked for me]. But they are expensive, and if it was somebody like me in my financial situation, I would probably say to them, like, "If you can wait a little bit longer after you wake up," like those types of things. [ID Post-2489]
	I appreciate the free medication you gave me, and I am going to put it to good use. [ID Post-1525]
**Readiness to quit (*n*** **=** **11)**	**Uptake**
	Not [using the lozenge] as often as I probably should. But when I'm ready to quit I'll be using it because I have it, and I know it helps… Only when I'm ready. [ID Pre-555,018]
	I didn't have the will to do it myself. [ID Post-3042]
	I'd say [I started using the NRT] about three or four days [after receiving it. That's when I really got serious about putting the cigarettes down. [ID Post-4304]
	[I chose to use the NRT because] I've been smoking for a lot of years, and I couldn't do it on my own. [ID Post-677]
**Beliefs about nicotine & medication** **(*n*** **=** **6)**	**Uptake**
	I feel as if it's just kind of a crutch…You're going to continue to do it, you might as well just stop 100% at the beginning. [ID Post-2583]
	I figured why put nicotine in my body if I'm trying to get rid of nicotine [laughter]. That's how I feel about nicotine lozenges. I really didn't want to take medication at all. [ID Post-4082]
	I did not use any medication. I just kept focusing on getting it done… I just feel like if I start taking the gum or the patch or something, then I'm kind of just changing the addiction.. I'm afraid if I do something else, then I'm not going to want to stop doing it. [ID Post-7067]
**Smoke free situations** **(*n*** **=** **4)**	**Uptake**
	I'm planning on going out and buying lozenges. I had them for the plane ride I had to take, and they seemed to work really well on the plane. [ID Pre-555,003]
	But usually, if I have no choice, like if I'm in the hospital for some reason and I can't smoke, then just the patch does work because I'm not focused on not smoking. [ID Pre-555,018]
**Peer narratives about NRT** **(*n*** **=** **8)**	**Uptake**
	And everyone I've talked to, nobody liked [the lozenge]. Nobody likes it. I've talked with a few people that like the gum. [ID Post-1639]
	My mom told me the nicotine lozenge burns her throat, so I never went that way. [ID Pre-555,056]
	**Consistent Use**
	The patch I would just take it off and then have a cigarette, and then put the patch back on. Because someone tried to [inaudible], if you put the patch on, you're going to have a heart attack. [ID Pre-555,038]
	**Persistence**
	I remember I did the patch and when I had done it, it was like, “Oh no, you can't smoke. You'll have a heart attack.” That was actually a long time ago. I just thought you still couldn't. [ID Post-2738]
	Yeah, I did [have concerns about the safety of the patch], that was why I took it off because I was seeing all these different stories like I wasn't very informed…Yeah, just from hearing from friends and just people around like in school. [ID Pre-555,004]
**Information gaps (*n*** **=** **10)**	**Uptake**
	[I waited] probably a week [to start using the NRT]. I'm not sure. I didn't know how to—maybe I was a little bit confused when to start using them even though it did say to start using them, I think, a week before or—I'm not sure. [ID Post-4199]
	I mean, there were a couple of different questions that I had and they were able to address it in terms of side effects [ID Post-4522]
	I knew what was in [the NRT]. I knew it was controlled. I knew it was prescribed. I knew it was recommended from doctors, so it made it feel safe. [ID Pre-55,022]
	So, it's a lot to do with fear too in what they're going to deal with like withdrawals or things like that. So, there's a lot of fear involved. If people were more informed, then it would be a lot better for them. And they'd feel like they're more secure in their decision and they can do it. But without all this information, things like that, people just—they don't try it. [ID Pre-555,067]
	[It was helpful when the doctor] went through this is how you do it, this is what you do with smoking, and this is how people have used it. This is how people are successful with it. [ID Pre-555,036]
	**Consistent Use**
	Because I tried the patches, and I didn't think you could smoke with the patches on. Then I found out you could. So, I did. I mean, I didn't smoke a lot. I tried not smoking at all and that wasn't working. Then I didn't wear it. [ID Post-2738]
	I've heard that you can't stop [the vaporizer] for long, something they're calling a hormone, so I mean if there was information about [safety of the vaporizer] I'd love to hear something about that. [ID Pre-555,003]
	Just make sure that people understand how much they can use at one time depending on how much they smoke. [ID Pre-555,018]
	**Persistence**
	The [patches] that I was getting prescribed were falling off my arm and they wouldn't last a whole day. Nobody's really helped me out with the medication use.... nobody's told me what to do or how to use it, or—so I haven't really had much guidance. Just a few minutes if the pharmacists took me aside for a minute… they never ask me about the patch and that stuff. So, I don't know, there wasn't much help for me out there in that way, so I did it on my own. [ID Pre-555,033]
	I don't think people know too much information like how the patch works....I did [have concerns about the safety of the patch], that was why I took it off because I was seeing all these different stories like I wasn't very informed…I think people just need to be more educated on how to use the different things to quit. [ID Pre-555,004]
**Incorporation of NRT into a routine** **(*n*** **=** **7)**	**Consistent Use**
	Every time I got in the car I had a cigarette. And then when I did quit, chewing the gum really did help. Especially in the car. [ID Pre-555,022]
	The patch I used every day. The medication I used, at first, kind of on a schedule. But then I just did it when I needed it. That's what I'm doing now. When I feel I need it. [ID Post-3240]
**Oral fixation** **(*n*** **=** **7)**	**Persistence**
	I think something that would've played a huge role for me, is—again, I enjoy the oral fixation, that hand-to-mouth. So giving myself—other than chewing gum and sucking on a piece of candy, which is common knowledge—well, at least for most people it's common knowledge. That really doesn't work for me. [ID Post-2544]
	I feel like the combination of the patch with the lozenge, the one-two combo, really was, I think, what helped because when you smoke, you have like an oral fixation thing… Last time, I used the patch…and I think it didn't address some of the physical urges that happen about smoking, right? [ID Post-4522]
**Disliked taste** **(*n*** **=** **10)**	**Persistence**
	I'm going to start using the patches because the gum tastes nasty… The lozenges taste nasty. [ID Pre-1525]
	The lozenges I didn't really like it [ID Post-4339]
**Side effects** **(*n*** **=** **4)**	**Persistence**
	The patch, like I said, gave me nightmares. [ID Pre-555,056]
	The patch itself, burned me. So that's why I didn't use the patch again. [ID Post-2544]
**Perceived effectiveness (*n*** **=** **12)**	**Persistence**
	On a daily basis for about six weeks. And at that point I noticed no improvement. So I've got to stop it. [ID Pre-555,015]
	I mean, when I got a craving or an urge, or some sort of panic attack about cigarettes, or something like that—I'm teasing. I said, “I can rely on these.” And physically, I'm sure they helped me a little bit. And mentally, they helped me a good amount. So it was like a substitute. It could have been a placebo, but it was helpful. [ID Post-4304].
	I tried the patch and the lozenges. That worked for about a day [laughter]… it might've been a little more beneficial if I'd been offered some alternatives like the Chantix or the nasal sprays that they have or whatever other kind of medically assisted treatment is available to people. [ID Post-2544]

⁎ Frequencies (n) reflect number of participants reporting each theme.

#### 3.2.1 Uptake

Six themes emerged as facilitators and/or barriers to uptake of NRT.

**Cost:** Several participants reported that the high cost of over-the-counter NRT felt inaccessible and prevented them from purchasing NRT. Participants reported that the free provision of NRT was a facilitator to their use.

**Readiness to quit:** Two uptake processes emerged depending on participant motivation and readiness to quit. Some participants felt that NRT increased self-efficacy and was the support they needed to make a quit attempt. *“I didn't have the will to do it myself,”* (ID Post-3042). Others, contrastingly, reported that they would not start using NRT until they felt ready to quit*.*

**Nicotine and medication concerns:** Many participants said they did not want to continue to rely on nicotine, which prevented NRT uptake. *“I feel as if it's just kind of a crutch… you might as well just stop 100% at the beginning,”* (ID Post-2583). Some participants reported that they “*don't like taking medication*,” (ID Post-1692).

**Smoke-free situations**: A consistent facilitator to NRT uptake were situations in which participants said that they were unable to smoke combustible cigarettes. “*I'm planning on going out and buying lozenges. I had them for the plane ride… and they seemed to work really well”* (ID Pre-555,003).

**Peer narratives about NRT**: Some participants mentioned hearing stories about NRT from friends or family, and these stories affected uptake. Positive stories were facilitators, while negative stories, especially surrounding safety, were barriers to uptake. ***“****No [I did not use the NRT] … people were saying like, if you smoke with the patch, it'll get worse … Like the side effects. So, I just, kind of, felt scared”* (ID Pre-555,004).

**Information needs**: Some participants mentioned uncertainty about when or how to start using NRT. This uncertainty was a barrier to uptake. “*But without all this information [on NRT], things like that, people just—they don't try it,”* (ID Pre-555,067).

#### 3.2.2 Consistent use

Three themes impacted participants’ consistent use of NRT as prescribed.

**Incorporation into a routine**: Using NRT as part of daily regimen was a facilitator to consistent use.

**Peer narratives about NRT:** While most of the stories about peer narratives were in reference to uptake or persistence, one participant did report intermittent use because of hesitation, primarily due to safety concerns, stemming from these stories. This was a barrier to their consistent use of the NRT. *“I would just take [the patch] off and then have a cigarette, and then put the patch back on. Because someone tried to [inaudible], if you put the patch on, you're going to have a heart attack”* (ID Pre-555,038).

**Information needs**: Information from a clinician was described as facilitating consistent use and increased confidence in using NRT. “*make sure that people understand how much they can use at one time depending on how much they smoke*,” (ID Pre-555,018). Participants who mentioned a lack of counsel on NRT expressed more uncertainty about how to use it correctly.

#### 3.2.3 Persistence

Six themes impacted persistence of NRT use.

**Peer narratives about NRT**: Peer anecdotes about NRT impacted persistence. Participants who were concerned about side effects or dangers of NRT reported stress when using it, which caused them to stop using the NRT.

**Information needs**: Participants reported that a lack of information on NRT safety or proper use caused them to stop using the medication. *“Nobody's told me what to do or how to use it… there wasn't much help for me out there in that way, so I did it on my own”* (ID Pre-555,033).

**Disliked taste**: Participants reported stopping use due to disliking the taste of the NRT gum or lozenge. The patch was an alternative to this.

**Side effects**: A few participants who had experienced side effects to NRT, such as a skin rash or nightmares from the patch, reported this made them discontinue use.

**Alleviation of oral fixation**: Many participants mentioned the oral fixation attached with smoking cigarettes. When a participant felt that NRT helped with this fixation, such as the ritual of putting a lozenge in one's mouth, they reported this facilitated persistence. Other participants reported that the patch did not support them in this way.

**Experienced effectiveness**: Patients reported that their experienced effectiveness of NRT influenced how long they continued with the NRT. Participants frequently reported that upon noticing no improvement in ability to abstain from cigarettes, they often stopped NRT use.

“I said, ‘I can rely on these.’ And physically, I'm sure they helped me a little bit. And mentally, they helped me a good amount…It could have been a placebo, but it was helpful” (ID Post-4304).

## 4 Discussion

Results from these interviews illuminate the variability of facilitators and barriers to NRT along three different adherence behaviors: uptake, consistent use, and persistence. This is one of the first studies to describe facilitators and barriers to NRT use across a dynamic adherence pathway. While some themes are related to one stage of the adherence pathway (e.g., side effects influenced persistence), other themes were represented over all three aspects of the adherence pathway, sometimes in varying fashions. For example, information needs were pertinent to each behavior, but the specific nature of those information needs varied between the three behaviors. Our study also highlights the importance of peer influence on NRT adherence. Consistent with other studies, we found that unaffordability of NRT, side effects, and a negative view of nicotine were barriers to use (Shiffman et al., 2008; Yingst et al., 2015), and that confidence in NRT safety is a facilitator to adherence (Kim et al., 2019). Also, some themes that have been described in other studies on NRT adherence, such as forgetting to use NRT or not feeling that NRT was necessary (Yingst et al., 2015), were not represented within our sample.

Importantly, many of the barriers and facilitators we identified are modifiable and could be addressed with messaging tailored to the patient's knowledge, beliefs, and stage in their quit journey. For example, cost barriers indicate the importance of considering socioeconomic and insurance status when prescribing or recommending NRT. In Massachusetts, Medicaid plans cover NRT. Almost half of our sample received Medicaid benefits, but the results from these interviews suggest that many patients may not know about their free or low-cost options. Improving awareness of their insurance benefits, such as through informational handouts, may increase NRT uptake. The emergence of cost as a factor impacting NRT adherence may also indicate that the situated-IMB model (Rivet Amico, 2011), which accounts for socioeconomic contexts, would be a useful model for future research into these adherence processes.

Other barriers to uptake were related to behavior, beliefs, and attitudes. Readiness to quit may be a modifiable factor; for some patients, offering NRT may be a nudge to take action while others may benefit from recommendations to consider starting NRT prior to a quit date. Indeed, one study showed that proactively providing a sample of NRT increases medication use and quit attempts among both unmotivated and motivated smokers (Carpenter et al., 2020). Additionally, hesitation about using nicotine for smoking cessation, while a strong barrier, could be modified. Some participants reported concern over continuing to rely on nicotine [Table 3]. Negative beliefs about nicotine for smoking cessation have been demonstrated previously (McDaid et al., 2021; Morphett et al., 2015) where some individuals who smoke believe NRT may be as harmful as cigarettes (Shiffman et al., 2008). These results show that negative views of nicotine for smoking cessation are present in our population. Providing information about NRT safety may impact smoking outcomes. This could be done in ways that do not require additional provider time, such as informational posters or product inserts. Regarding consistent use of NRT, incorporating NRT into one's routine is a modifiable factor; brief advice that includes behavioral skills support, such as tips for incorporating NRT into routines and information about the onset of nicotine delivery with patches and low potential for addiction, may improve consistent use.

Some barriers to NRT adherence were themselves non-modifiable, but with mitigatable impacts. For example, side effects and disliking the taste of NRT lozenge were barriers. However, acknowledgement of these possibilities and the timely offer of another product option could improve use. Side effects came up during interviews, but not often. They are expected among a minority of NRT users (Mills et al., 2010). Providing a handout with the list of potential side effects, and actions to take if those side effects arise, could mitigate their impact on discontinuation by treating mild rash, altering lozenge use to minimize mouth complaints, or offering alternative pharmacotherapy to patients motivated to quit. Additionally, while the hand-to-mouth oral fixation may not be modifiable itself, the impact of this fixation could be mitigated by encouraging use of dual short and long-acting NRT including an oral product like gum or prescription inhaler. Considering our findings that patients may stop NRT use abruptly after noticing no immediate relief in cravings, providers or counselors should consider intervening (such as recommending dual forms of NRT) while patients are still using NRT, in case the patient dislikes the taste, experiences side effects, or does not feel that the NRT meets their behavioral needs.

Certain themes were cross-cutting, affecting uptake, consistent use, and persistence. Two modifiable factors overlapped significantly and were consistently found along the entire NRT adherence pathway: peer narratives about NRT and NRT information needs. Our findings suggest that there are significant gaps in knowledge surrounding NRT safety, which are sometimes filled by negative anecdotes about NRT from peers. This misinformation may prevent people from initiating use, using NRT in the way it is intended, or continuing use for the recommended duration. For example, some people stopped using the patch, or never started, because they heard from others that smoking with the patch may cause a heart attack. It is challenging to shift misperceptions and attitudes around smoking and tobacco cessation (Morphett et al., 2016), but some interventions have shown success (Sharma-Kumar et al., 2021). Asking patients what they have heard about NRT may equip clinicians to efficiently address barriers arising from those narratives. Alternatively, digital media and public health campaigns could target these myths about NRT. Interviews also highlighted that information needs vary over the course of the adherence pathway: participants expressed confusion about when or how to begin NRT use (uptake), how much NRT to use (consistent use), and insecurity about NRT safety of continued use (persistence). Since the information needs and anecdotes heard from peers often overlapped in content, it may be that those who are less informed about NRT safety are particularly reliant on peer narratives.

Many of the information needs detailed in the interviews could be addressed with counseling or additional resources, such as handouts or text message programs. Participants identified their providers as a trusted source of information. One participant noted that they felt comfortable using the NRT because they “knew it was prescribed.” It may be helpful for someone in the office or pharmacy to provide materials when a new prescription is written that describes the safety of the NRT, its details of use, and when to call the office. Creating opportunities for patients to share concerns throughout the quit journey, not just prior to NRT prescription, may be needed.

### 4.1 Limitations

There are several limitations in this analysis. It is a secondary analysis of interviews conducted for two pilot studies to inform the design of a text message intervention. Our qualitative study is exploratory in nature and reflects the experiences of our small sample of participants from one healthcare system. We asked participants about their most recent use of any form of NRT (gum, patch, lozenge, inhaler) and did not examine the barriers and facilitators to medication adherence for each form individually. Notably, 83% of our participants were white, which is consistent with the population of patients who smoke in our primary care network, but may not produce in-depth understanding of adherence processes for diverse populations (El-Toukhy et al., 2016). Nevertheless, some of the barriers to NRT adherence discussed here have been reported in non-white populations, suggesting that these barriers may traverse demographics and countries (Yuke et al., 2018). Lastly, the study took place in a state with the highest rate of insurance coverage in the nation (United States Census Bureau, 2020) and a generous insurance benefit for smoking cessation in the state Medicaid program.

## 6 Conclusion

Our results describe variation in the barriers and facilitators to NRT uptake, adherence, and consistent use. NRT adherence is not static, and the variability in adherence processes shown here demonstrates a need to recognize where people are in their quit journey, their experience using NRT, and the stories they have heard about NRT as a smoking cessation aid. The results suggest that engaging with patients about their perceptions and knowledge, readiness to quit, and their socioeconomic status when advising them on the use of NRT for smoking cessation could improve the real-world effectiveness of these medications through improved adherence. Additionally, public health campaigns could target common myths about NRT and provide information about potential side effects, methods for use, and insurance coverage through informational handouts, posters, digital media campaigns, or product inserts. Considering the common barriers to NRT uptake, including affordability, readiness to quit, NRT safety, and patients’ understanding of how different forms of NRT work and should be used, could increase the effectiveness of public health interventions. Those barriers which were pervasive across all three levels of adherence—peer narratives and informational needs—may represent particularly impactful factors to intervene upon to improve smoking outcomes.

## Contributors

All authors provided critical feedback to shape the conceptualization of the research, analysis and manuscript.

Styklunas, GM: Project administration; original draft preparation, writing and editing; analysis of interview transcripts using analytic memos and process coding.

Shahid, N: Project administration; conduct of interviews; coding structure and theme generation, editing

Park, ER, Ph.D.: Writing review, editing and advising, contributed to conceptualization of the study design.

Haberer, JE, M.D.: Writing review, editing and advising, contributed to conceptualization of the study design.

Rigotti, NA, M.D.: Writing review, editing and advising, contributed to conceptualization of the study design.

Howard, Se: Writing review, editing and advising, conduct of interviews

Kruse, GR: Original draft preparation, writing and editing; funding acquisition; conduct of interviews; coding structure and theme generation; analysis of interview transcripts using analytic memos and process coding, led study conceptualization and design.

All authors have read and agreed to the published version of the manuscript.

## Declaration of Competing Interest

GRK has a family financial interest in Dimagi, Inc.

NAR has consulted with Achieve Life Sciences, regarding an investigational smoking cessation medication, and receives royalties from UpToDate for review of smoking cessation treatments.

JEH consults for Merck and the CDC.
